# Different types of anemia in patients with chronic kidney disease

**DOI:** 10.12669/pjms.41.7.11398

**Published:** 2025-07

**Authors:** Syed Ahmad Shah, Muhammad Bilal, Yaseen Khan, Miaz Fareezuddin

**Affiliations:** 1 Syed Ahmad Shah, MBBS, FCPS. Medical Officer, Lady Reading Hospital, Peshawar, Pakistan; 2 Muhammad Bilal, MBBS, FCPS, MRCPI, FRCPI, CHPE, CHR. Associate Professor, Medicine, Lady Reading Hospital, Peshawar, Pakistan; 3 Yaseen Khan, MBBS, FCPS, MRCPI, FRCPI. Professor, Medicine, Lady Reading Hospital, Peshawar, Pakistan; 4 Miaz Fareezuddin, MBBS, MPH, MCPS. Medical Officer, Federal Public Service Commission, Islamabad, Pakistan

**Keywords:** Anemia, Chronic kidney disease, Cross-Sectional studies, Hypochromic, Hyperchromic

## Abstract

**Objective:**

This study aimed to assess the prevalence of different anemia patterns in CKD patients.

**Methods:**

This cross-sectional study was carried out at the Outpatient Department of General Medicine, Lady Reading Hospital, Peshawar from 1st July to 31^st^ December, 2023. Non-probability consecutive sampling was employed. Demographic data was collected and anemia was evaluated through Complete Blood Counts for each participant. Statistical analysis was performed using SPSS version 23.

**Results:**

The study included a cohort of 209 participants with an average age of 40.78 ± 6.37 years and a mean BMI of 26.12 ± 4.11 kg/m². Among the participants, 72.2% were male and 27.8% were female. The most common CKD patterns were normocytic (41.1%), hypochromic microcytic (32.5%), and hypochromic macrocytic (26.3%). The majority of participants (78.9%) had experienced CKD for over one year. No significant association was found between the types of anemia in CKD and gender, underlying cause of CKD, age, BMI, or duration of the disease.

**Conclusion:**

Our study highlights the prevalence of various types of anemia in chronic kidney disease (CKD) among participants, with normocytic anemia emerging as the most prevalent, followed by hypochromic and hyperchromic patterns. Additionally, longitudinal studies could provide insights into the progression and management of anemia in CKD patients over time.

## INTRODUCTION

The preservation of kidney health is crucial for the overall well-being of individuals due to the vital role of the kidneys in eliminating harmful waste products from the body. Accumulation of these waste products can have detrimental effects on human health.^1^,^2^ Chronic kidney disease (CKD) is characterized by a prolonged decline in kidney function lasting more than three months, evidenced by a glomerular filtration rate (GFR) lower than 60 mL/min per 1.73m² and observable structural alterations in kidney tissue.^2^ Anemia is a frequent complication of CKD, significantly impacting the quality of life and increasing the risk of morbidity and mortality.^3^ Complications of Anemia in CKD includes; Hypoxia, Cardiorenal Anemia Syndrome (described by Van Der Putten K et al., this syndrome involves a cycle where decreased kidney function leads to reduced EPO production and anemia.^4^

Severe anemia causes compensatory left ventricular hypertrophy (LVH), precipitating chronic heart failure (CHF), further impairing renal function), Cardiovascular Disease, Erythropoietin Resistance (many CKD patients do not respond to erythropoietin, with iron deficiency and inflammation being significant predictors of resistance).^4^-^6^ The causes of anemia in CKD are multifaceted and include reduced production of endogenous erythropoietin (EPO), absolute or functional iron deficiencies, and inflammation leading to elevated levels of hepcidin, among other factors.^7^,^8^ In CKD patients, erythropoietin (EPO) levels are inadequately low relative to the degree of anemia, worsening as the estimated glomerular filtration rate (eGFR) falls below 30 ml/min per 1.73 m².^9^ Various types of anemia are observed in CKD patients, including hypochromic microcytic anemia, normochromic normocytic anemia, and mixed patterns of anemia.^10^ According to a study by OH Miah et al., normochromic normocytic anemia is the most prevalent form, observed in 66% of CKD patients, while hypochromic microcytic anemia occurs in 22%.^11^ The Dialysis Outcomes Practice Pattern Study (DOPPS), conducted across multiple countries, reported that hemoglobin levels below 11 g/dL are associated with increased hospitalization and mortality in CKD patients.^12^ Further, the National Health and Nutrition Examination Survey (NHANES) data from 2007–2008 and 2009–2010 indicated that anemia is twice as prevalent in CKD patients compared to the general population (15.4% vs. 7.6%). The prevalence of anemia increases with CKD progression, from 8.4% at stage one to 53.4% at stage five. Similar findings were reported by the CKD Prognosis Consortium, which also noted a higher prevalence of anemia in diabetic patients, independent of eGFR and albuminuria.^13^

Chronic kidney disease (CKD) often leads to anemia, a condition commonly managed with oral or intravenous iron supplements and erythropoiesis-stimulating agents (ESAs). However, these treatments can have associated risks and may not always be effective. CKD patients primarily undergo hemodialysis, with peritoneal dialysis and renal transplantation as alternative options.^14^,^15^ Despite the prevalence of anemia in CKD, limited research exists on the frequency of various anemia patterns in local populations. This study aimed to determine the frequency of various patterns of anemia in patients with chronic kidney disease, offering valuable data for future research and improved patient management.

## METHODS

This descriptive cross-sectional study was conducted in the Department of Medicine at Lady Reading Hospital, Peshawar from 1^st^ July to 31^st^ December, 2023. Using the WHO sample size formula, a sample size of 209 was determined based on a 2% proportion of macrocytic anemia in CKD patients from previous literature, with a margin of error of 1.9% and a confidence level of 95%. Non-probability consecutive sampling was employed. Participants aged 20 to 50 years of both genders with a history of chronic kidney disease for at least six months and presenting with anemia were included. Exclusion criteria comprised patients with genetic hemoglobin diathesis, bleeding diathesis, recent blood transfusions (within the last six months), recent iron supplement intake (within the last six months), and a history of malignancy.

### Ethical Approval:

It was obtained from the hospital’s research review board with Ref. No. 796/LRH/MTI on dated 22^nd^ June, 2023. Eligible patients were enrolled from the OPD of Medicine, and informed consent was obtained from all participants.

Information regarding participants age, gender, BMI, and CKD duration were recorded through designed questionnaire. Detailed medical history was obtained and examinations were conducted to identify signs of anemia. Blood samples were collected from study participants following standard procedures. Venous blood samples from the antecubital vein using sterile techniques by a trained phlebotomist. The collected blood was then transferred into ethylenediaminetetraacetic acid (EDTA) tubes to prevent clotting and then sent to the laboratory for CBC analysis. The CBC included measurements of red blood cells (RBCs), white blood cells (WBCs), platelets, hemoglobin, hematocrit, and RBC indices. Normal reference ranges were used for interpretation. Peripheral smears were made by wedge technique and stained. Blood smears were evaluated by hematologist and type of anemia was determined.

### Statistical analysis:

Data was recorded using a specially designed proforma. Statistical analysis was performed using IBM SPSS version 23. Frequencies and percentages were calculated for qualitative variables, including gender, cause of CKD, and anemia pattern. Means and standard deviations were calculated for quantitative variables such as age, BMI, and CKD duration. Effect modifiers like age, gender, BMI, CKD cause, and CKD duration were controlled through stratification. Post-stratification chi-square tests were applied, with a p-value of ≤0.05 considered statistically significant.

## RESULTS

The study included a total of 209 participants. The average age of the participants was 40.78 ± 6.37 years. The mean Body Mass Index (BMI) was 26.12 ± 4.11 kg/m². The average duration of chronic kidney disease (CKD) among the participants was 2.15 ± 0.86 years. About 72.2% (n=151) of the participants were male and 27.8% (n=58) were female. The primary causes of CKD among the participants were Diabetes, accounting for 57.9% (n=121) of the cases, followed by hypertension about 20.1% (n=42), autoimmune causes about 12.0% (n=25), and other causes at 10.0% (n=21). The majority of the participants were in the 41 to 50 years age category about 58.9% (n=123), 31 to 40 years comprised 34.9% (n=73), while those aged 18 to 30 years were about 6.2% (n=13). The majority of the participants were categorized as overweight (42.6%), followed by those with a normal BMI (39.2%). Obesity was observed in 16.7% of the participants, while a small proportion (1.4%) were underweighted.

**Fig.1:**
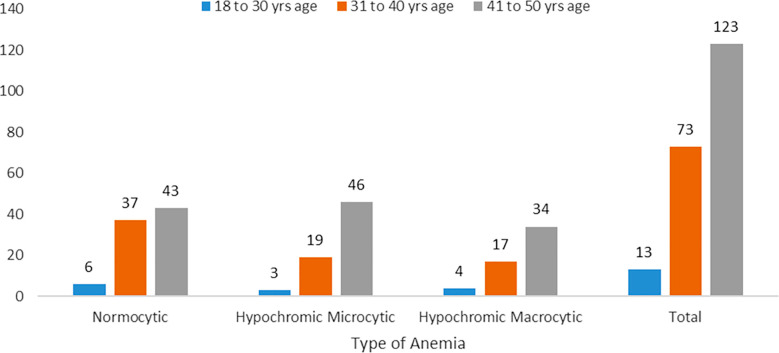
Type of Anemia in different Age Groups.

Analysis of the anemia type among CKD patients revealed that Normocytic anemia was the most prevalent, observed in 41.1% (n=86) of the total 209 cases. Hypochromic microcytic anemia accounted for 32.5% (n=68) of the cases, while macrocytic anemia was present in 26.3% (n=55) of the cases. Further analysis revealed that among 206 cases, normocytic anemia was observed in 63 males and 23 females, hypochromic microcytic anemia in 51 males and 17 females, and hypochromic macrocytic anemia in 37 males and 18 females. The distribution of anemia types across genders. The p-value of 0.613 indicates no statistically significant difference in type of anemia in CKD between males and females, illustrated in Table-I. Fig.1 presents the distribution of type of anemia in CKD across various age groups among the participants. The p-value of 0.242 suggests no statistically significant difference in type of anemia in CKD patients across different age groups.

**Table-I:** Gender of CKD patients with different types of anemia.

Gender	Normocytic Anemia	Hypochromic Microcytic Anemia	Hypochromic Macrocytic Anemia	Total	p-value
Male (n=151)	63 (41%)	51 (34%)	37 (24%)	151	0.613
Female (n=58)	23 (40%)	17 (29%)	18 (31%)	58	
Total	86	68	55	209	

The analysis of relationship between BMI categories and type of anemia in CKD presented in Table-II, revealing that the majority of normocytic anemia cases were found in the overweight category. Specifically, there were 43 normocytic cases, 28 hypochromic microcytic cases, and 18 hypochromic macrocytic cases among overweight participants. Overall, there were 86 normocytic, 68 hypochromic microcytic, and 55 hypochromic macrocytic cases across all BMI categories. The p-value of 0.492 indicates no statistically significant difference in type of anemia in CKD among the different BMI categories. Table-III analyzes the relationship between the cause of CKD and type of anemia present, showing that diabetic patients were diagnosed with all three types of anemia. Specifically, diabetes was associated with 51 normocytic, 34 hypochromic microcytic, and 36 hypochromic macrocytic cases. In comparison, hypertension accounts for 14 normocytic, 22 hypochromic microcytic, and six hypochromic macrocytic cases. Autoimmune causes were linked to 12 normocytic, 8 hypochromic microcytic, and five hypochromic macrocytic cases. The p-value of 0.255 indicates no statistically significant difference in type of anemia in CKD based on the cause of CKD.

**Table-II:** BMI Categories association with type of Anemia in CKD.

BMI Categories	Normocytic Anemia	Hypochromic Microcytic Anemia	Hypochromic Macrocytic Anemia	Total	p-value
Underweight (n=3)	1 (33%)	0 (0%)	2 (77%)	3	0.492
Normal (n=82)	27 (33%)	29 (35%)	26 (32%)	82	
Overweight (n=89)	43 (48%)	28 (32%)	18 (20%)	89	
Obese (n=35)	15 (43%)	11 (31%)	9 (26%)	35	
Total	86	68	55	209	

**Table-III:** Association of causes of CKD with type of Anemia.

Causes of CKD	Normocytic Anemia	Hypochromic Microcytic Anemia	Hypochromic Macrocytic Anemia	Total	p-value
Diabetes (n=121)	51 (42%)	34 (28%)	36 (30%)	121	0.255
Hypertension (n=42)	14 (34%)	22 (52%)	6 (14%)	42	
Autoimmune (n=25)	12 (48%)	8 (32%)	5 (20%)	25	
Other (n=21)	9 (43%)	4 (19%)	8 (39%)	21	
Total	86	68	55	209	

In Table-IV, the distribution of anemia types based on the duration of CKD among the participants. In participants with CKD, there were 86 total cases of normocytic anemia, with 22 cases lasting less than one year and 64 cases lasting more than one year. For hypochromic microcytic anemia, there were 68 total cases, with nine cases lasting less than one year and 59 cases lasting more than one year. For hypochromic macrocytic anemia, there were 55 total cases, with 13 cases lasting less than one year and 42 cases lasting more than one year. The p-value of 0.151 indicates no statistically significant difference in anemia types based on CKD duration.

**Table-IV:** Association of CKD duration with type of Anemia.

Duration of CKD	Normocytic Anemia	Hypochromic Microcytic Anemia	Hypochromic Macrocytic Anemia	Total	p-value
Less than one year (n=44)	22 (50%)	9 (20%)	13 (30%)	44	0.151
More than one year (n=165)	64 (39%)	59 (36%)	42 (25%)	165	
Total	86	68	55	209	

## DISCUSSION

The demographic data analysis in our study closely resemble to the study conducted by Ali et al. (2022), where the mean age was 43.51 ± 5.652 years, and the mean duration of CKD was 4.36 ± 3.712 years.^16^ Our study comprised 72.2% males and 27.8% females among the 209 participants which is in line with the study by Ali et al., which included a total of 300 patients, 139 were males, and 161 were females.^16^

In our study, the primary causes of chronic kidney disease (CKD) were diabetes (57.9% of cases), hypertension (20.1%), and autoimmune causes (12.0%). This is contrary to Khan et al., who reported glomerulonephritis as the most common cause of anemia in CKD patients, accounting for 63.52% of cases.^17^ Similarly, El-Achkar et al. found a significantly higher frequency of anemia in diabetic patients compared to non-diabetic individuals.^18^ Anees M et al. also highlighted a strong association between anemia and diabetes mellitus in CKD patients, with 61.64% of their patients having diabetes mellitus.^19^

In our study, 41.1% of CKD cases were normocytic, 32.5% hypochromic microcytic, and 26.3% hypochromic macrocytic. This aligns with OH Miah et al., who found 66.0% of CKD patients had normochromic normocytic anaemia and 22.0% had hypochromic microcytic anemia.^11^

In our study, 58.9% of participants were in the 41 to 50 years age group, 34.9% were in the 31 to 40 years group, and 6.2% were in the 18 to 30 years group. Moreover, we found no significant association between age categories and anemia in CKD patients (p = 0.242). While Khan et al. reported that 14.37% of their study participants were aged 20-50 years, and 34.25% were aged 50 and above.^17^

Furthermore, Ravani et al., 2020, reported a significant association between age and anemia in CKD, stating that the prevalence of CKD increases with age.^20^ Yuniarti emphasized the relationship between anemia and CKD, noting that the prevalence of anemia is higher in older populations, particularly those aged 75 years and above.^21^

Additionally, our study found no significant association between gender and anemia in CKD (p = 0.613). In contrast, studies have shown that anemia is more prevalent in female CKD patients compared to males.^22^,^23^

The association between body mass index (BMI) and anemia in chronic kidney disease (CKD) patients is complex. In the current study majority of normocytic anemia cases were found in the overweight category. Specifically, there were 43 normocytic, 28 hypochromic microcytic, and 18 hypochromic macrocytic cases among overweight participants. Overall, there were 86 normocytic, 68 hypochromic microcytic, and 55 hypochromic macrocytic cases across all BMI categories. Also, there was no statistically significant difference in type of anemia in CKD among the different BMI categories. Literature confirms that higher BMI is linked to improved hemoglobin levels in advanced CKD stages.^24^ However, underweight men with abnormal kidney function have a higher risk of anemia compared to normal-weight men, while overweight men show a lower risk.^25^ Obesity is associated with increased odds of CKD in Asian populations.^26^ A meta-analysis identified several risk factors for anemia in CKD patients, including female sex, stage 5 CKD, and BMI ≥30 kg/m^2^.^27^ The relationship between BMI and anemia in CKD patients appears to be influenced by factors such as CKD stage, sex, and kidney function.

Based on our findings, we recommend enhanced screening and monitoring for anemia in CKD patients, especially those with diabetes and hypertension, to enable early detection and timely management. Treatment plans specific to the types of anemia could improve patient outcomes. Additionally, educating patients on the importance of managing CKD and its complications, including regular check-ups and recognizing anemia symptoms, is crucial for improving health outcomes.

### Strength of this study:

The strength of this study lies in its local setting and comprehensive data collection, offering new insights into anemia patterns among CKD patients in Peshawar, a population underrepresented in current literature. By identifying normocytic anemia as the most prevalent type and finding no significant association between anemia types and common demographic or clinical variables, the study challenges existing assumptions and emphasizes the need for region-specific clinical approaches. Conducted in a tertiary care hospital with a well-maintained patient database, the findings reflect real-world CKD profiles and have direct clinical relevance for screening and management practices. This study highlights the importance of routinely monitoring anemia in diabetic and hypertensive CKD patients, regardless of age or gender. Future research should include biochemical profiling and longitudinal designs to explore causal relationships and the progression of anemia in diverse CKD populations.

### Limitations:

Despite the valuable insights provided, this study opens up several avenues for future research. Longitudinal studies that track the progression of anemia types in CKD patients over time would be invaluable. This would allow researchers to observe how anemia evolves as CKD progresses and how different treatments affect anemia outcomes. Additionally, there is a need for multi-center studies to enhance the generalizability of these findings. Randomized controlled trials focused on the effectiveness of anemia treatment regimens for the different anemia patterns observed in CKD patients would also help establish more specific management guidelines.

## CONCLUSION

Our study determined the frequency and types of anemia in patients with chronic kidney disease (CKD). We observed all types of anemia among the patients with CKD, in which normocytic anemia was the most prevalent, followed by hypochromic microcytic and hypochromic macrocytic types. The leading causes of CKD in our study were diabetes, hypertension, autoimmune conditions, and other factors. Gender and age did not show significant associations with CKD patterns in our study, contrary to some previous findings. Our findings contribute to the understanding of anemia types in CKD patients within our population. Additionally, longitudinal studies could provide insights into the progression and management of anemia in CKD patients over time. By optimizing management strategies, healthcare providers can strive to improve the quality of life and clinical outcomes for individuals living with CKD-associated anemia.
